# Pterygomandibular raphe invasion as a novel grading and prognostic factor for squamous cell carcinoma of buccal mucosa: a retrospective study with propensity score matching

**DOI:** 10.1007/s10147-025-02821-2

**Published:** 2025-07-11

**Authors:** Ryoji Yoshida, Riu Lin, Keisuke Yamana, Takeshi Obayashi, Hisashi Takeshita, Kosuke Shinohara, Kenta Kawahara, Masatoshi Hirayama, Nozomu Takahashi, Akiyuki Hirosue, Masanori Shinohara, Hideki Nakayama

**Affiliations:** 1 Department of Oral and Maxillofacial Surgery, Faculty of Life Sciences, Kumamoto University, Honjo 1-1-1, Chuo-Ku, Kumamoto, 860-8556 Japan; 2 Division of Oral and Maxillofacial Surgery, Minamata City General Hospital & Medical Center, Minamata, Japan; 3 Division of Oral and Maxillofacial Surgery, Kumamoto Kenhoku Hospital, Tamana, Japan; 4 Division of Oral and Maxillofacial Surgery, Kumamoto City Hospital, Kumamoto, Japan; 5 Division of Oral and Maxillofacial Surgery, Ariake Medical Center, Arao, Japan; 6 Itoh Dento-Maxilofacial Hospital, Kumamoto, Japan

**Keywords:** Squamous cell carcinoma, Buccal mucosa, Pterygomandibular raphe, Prognosis, Retrospective study, Propensity score matching

## Abstract

**Background:**

Squamous cell carcinoma of the buccal mucosa (SCCBM) is a prevalent malignancy of the oral cavity with high morbidity and mortality rates. The pterygomandibular raphe (PMR) connects the oral cavity, pharynx, and masticator space, serving as a key anatomical landmark during surgical resection. However, PMR invasion into SCCBM remains poorly understood. This study assessed the prognostic significance of PMR invasion in patients with resectable SCCBM.

**Methods:**

The study included 82 patients with SCCBM, excluding those with T4b, who underwent radical resection at Kumamoto University Hospital between 2000 and 2017. Patients were categorized into three groups based on PMR invasion patterns: non-contact, contact, and invasion. The relationship between PMR invasion and clinicopathological characteristics was analyzed using the Fisher’s exact test. The Kaplan–Meier method, log-rank test, and propensity score-matched analyses were used for survival analysis.

**Results:**

Contact or invasion into the PMR was significantly associated with advanced clinical T and N stages, clinical stage, endophytic growth pattern, high-grade invasion pattern, and poor tumor differentiation. Patients with PMR invasion had a higher proportion of recurrences in the buccal subcutaneous, mandibular, and masticator spaces. Univariate analysis indicated that contact- or invasion-type PMR invasion predicted worse overall survival (OS). The impact of PMR invasion on OS was confirmed using a propensity score-matched analysis.

**Conclusion:**

This study revealed that PMR invasion is a potential novel grading and prognostic factor for resectable SCCBM, with significance in planning the extent of SCCBM resection.

**Supplementary Information:**

The online version contains supplementary material available at 10.1007/s10147-025-02821-2.

## Introduction

Squamous cell carcinoma of the buccal mucosa (SCCBM) is a relatively uncommon form of oral cancer with varying prevalence across different geographical regions. Although it is less frequent in most parts of the world [1, 2], it has a higher incidence in certain Asian countries [3, 4]. Notably, Japan has reported a particularly low occurrence of this cancer type [5], which has resulted in a limited understanding of its clinicopathological features.

The buccal mucosa, the site of SCCBM development, is an important anatomical structure that lines the inner cheek and lips. It extends from the point where the lips meet to the attachment points at the alveolar ridge and pterygomandibular raphe (PMR) [6, 7]. The anatomical location and connections of the buccal mucosa contribute to the complexity of SCCBM. The PMR is a significant landmark in this region that serves as a connective tissue bridge linking the oral cavity to several important anatomical structures, including the mandible, masticator space, mesopharynx, and maxilla [8]. This intricate network of connections highlights the potential of SCCBM to affect multiple areas, and underscores the importance of thoroughly understanding and managing this cancer type. The unique anatomical features of the buccal mucosa, combined with the varying prevalence of SCCBM across different populations, emphasize the need for region-specific studies and tailored approaches for the diagnosis, treatment, and prevention of oral cancer.

Prognostic factors for SCCBM have been identified, including advanced age, more severe worst pattern of invasion (WPOI-5), and perineural infiltration [9]. However, the lack of agreement between studies on these factors emphasizes the need for additional research and standardization. The absence of established guidelines for incorporating these prognostic factors into treatment approaches and surgical techniques underscores the importance of developing comprehensive protocols for SCCBM management. This highlights the need for new grading systems and prognostic indicators to improve patient outcome. Although various prognostic factors have been recognized in SCCBM, the importance of PMR invasion remains insufficiently explored, particularly in guiding treatment strategies. This knowledge gap provides an opportunity to examine the potential impact of PMR invasion on patient prognosis and treatment outcomes. By focusing on this understudied aspect of SCCBM, more targeted and effective treatment approaches may be developed to improve the survival rates and quality of life of patients with this malignancy.

A recent study by Xu et al. revealed that PMR invasion occurs in approximately half of the SCCBM cases [10]. In addition, Otsuru et al. proposed a three-type classification system for PMR invasion and examined its clinical and prognostic implications in oral squamous cell carcinoma (OSCC) across various sites [11]. Despite these advancements, the clinical and prognostic significance of PMR invasion in resectable SCCBM remains unclear. Therefore, additional studies focusing specifically on resectable SCCBM are necessary to determine the relationship between PMR invasion and patient outcome. This may lead to more tailored and effective treatment protocols for this subset of patients with oral cancer.

This study aimed to examine the prognostic significance of PMR invasion in patients with resectable SCCBM with the goal of identifying a novel prognostic index for improving treatment planning.

## Materials and methods

This retrospective study involved patients with SCCBM who presented to the Department of Oral and Maxillofacial Surgery of Kumamoto University Hospital between January 1, 2000, and December 31, 2017. Patients with SCCBM who had no history of head or neck cancer, including oral cancer, and who had never undergone radical resection or any prior treatment were included. Conversely, this study excluded cases in which magnetic resonance imaging (MRI) could not be performed to assess PMR invasion, as discussed subsequently. Further, cases classified as cT4b were excluded, where MRI images clearly illustrated tumors invading the masticator space, pterygoid process, or skull base, or circumferentially encasing the internal carotid artery. This study was approved by the Ethics Committee of Kumamoto University (RINRI, 1928). This study was conducted in accordance with the ethical standards of the institutional and/or national research committee and the 1964 Helsinki Declaration and its later amendments. Owing to the retrospective nature of the present study, the requirement for informed consent was waived by the Ethics Committee of Kumamoto University. However, it guarantees participation in the study and the opportunity to refuse participation in an opt-out format. (RINRI1427). The cervical region was evaluated preoperatively using a combination of computed tomography (CT), MRI, positron emission tomography with CT (PET-CT), and ultrasonography. Cancer staging was conducted in accordance with the International Union Against Cancer (UICC) 7th edition [12], and tumor differentiation was ascertained according to the World Health Organization classification [13]. The pattern of invasion (POI) was defined as previously described [14]. The primary tumor was surgically resected in accordance with the findings of Ota et al. [15], considering the spatial relationship between the tumor and buccinator muscle. Neck dissection was performed when cervical lymph node metastasis was clinically suspected, or when cervical vascularization was required for reconstructive surgery. Postoperative treatment was considered if histopathological assessment revealed positive surgical margins of the primary tumor or extranodal extension (ENE) of the cervical lymph nodes. However, the decision to undertake the procedure was ultimately discussed with the patient and not performed on all patients who met the criteria. Three surgeons (M.S., H. N., and R.Y.), with meticulous attention paid to maintaining consistency in surgical techniques and procedures within the institution, performed all surgical procedures. The postoperative follow-up schedule was structured as monthly during the first postoperative year, bi-monthly during the second year, quarterly during the third year, and semiannually from the fourth year onward, concluding at 5 years postoperatively. In conjunction with the physician’s assessment, imaging modalities such as cervical echocardiography, MRI, and CT were conducted concurrently whenever feasible. The median observation period for this study was 41.5 months, with a range from a minimum of 3 months to a maximum of 60 months.

### Evaluation of PMR invasion

PMR invasion was evaluated on the basis of the findings of Otsuru et al. [11]. In brief, contrast-enhanced MRI (short TI inversion recovery, weighted imaging) was used to assess PMR invasion. The buccinatopharyngeus muscle was identified on the axial image, and the PMR was identified as the region extending from the pterygoid process to the mandibular molar (superior and inferior) and from the anterior mandibular branch to the pterygoid process (anterior and posterior). The diagnosis was established using MRI by a consensus of expert radiologists and oral surgeons (Supplementary Figure S1).

### Statistical analysis

Categorical data are presented as numbers and percentages, whereas quantitative data are presented as mean and standard deviation. Fisher’s exact test was used to compare categorical factors between groups. The significance of the association between the type of PMR invasion and clinicopathological factors was determined by calculating the *p* values. Survival analysis and cumulative local control rates were assessed using the Kaplan–Meier method and log-rank tests. Multivariate survival analyses were performed using Cox regression models to evaluate the predictive value of clinicopathological factors, including the invasion-type PMR for 5-year local control rate (LCR), disease-free survival (DFS), and overall survival (OS). The effects of selection bias were mitigated using propensity score matching (PSM). A propensity score-matched cohort for the non-contact and contact/invasion groups was constructed using 1:1 nearest neighbor matching with a caliper width of 0.2, followed by a logistic regression analysis calculation. A standardized mean difference of < 0.1 indicated adequate balance following PSM. Univariate analysis was performed to determine the potential prognostic factors. The factors identified in the univariate analysis were subsequently incorporated into the multivariate analysis to ensure model reproducibility. All *p* values were determined using two-tailed tests, and statistical significance was set at *p* < 0.05. All statistical analyses were performed using EZR (Saitama Medical Center, Jichi Medical University, Saitama, Japan), which is a graphical user interface for R (The R Foundation for Statistical Computing, Vienna, Austria) and more precisely a modified version of R commander designed to add statistical functions frequently used in biostatistics [16].

## Results

### Clinicopathological characteristics

Table 1 summarizes the clinicopathological characteristics of patients included in this study. This study included 82 patients with SCCBM, of whom 50 (61.0%) were male. In the clinical setting, 61 of the 82 patients (74.4%) were older than 65 years. Clinically suspected cervical lymph node metastases were present in 35 patients (42.7%). Clinical stage III and IVA tumors were detected in 40 patients (48.8%). The superficial, exophytic, and endophytic tumor varieties were equally distributed, with approximately 30% of cases in each group. Among the 82 individuals examined in the context of PMR invasion patterns, 53 patients (64.6%) were classified as non-contact type, 12 (14.6%) as contact type, and 17 (20.7%) as invasion type. Histopathologically, most tumors were classified as POI-3 (42 patients, 51.2%), followed by POI-1 and POI-2 (25 patients, 30.4%). Highly invasive tumor grade POI-4 was detected in 15 (18.3%) patients.

**Table 1:** The clinicopathological characteristics of 82 patients with squamous cell carcinoma of buccal mucosa

Characteristics	Total
	n = 82
Age (years)	
≦ 64	21 (25.6)
≧ 65	61 (74.4)
Sex	
Male	50 (61.0)
Female	32 (39.0)
Clinical growth pattern	
Superficial	29 (35.4)
Exophytic	27 (32.9)
Endophytic	26 (31.7)
cT	
1	12 (14.6)
2	47 (57.3)
3	8 (9.8)
4a	15 (18.3)
cN	
0	47 (57.3)
1	14 (17.1)
2b	20 (24.4)
2c	1 (1.2)
cStage	
I	13 (15.9)
II	29 (35.4)
III	11 (13.4)
IVA	29 (35.4)
Differentiation	
Well	59 (72.0)
Moderate	19 (23.2)
Poor	4 (4.9)
Pattern of invasion	
POI-1	2 (2.4)
POI-2	23 (28.0)
POI-3	42 (51.2)
POI-4	15 (18.3)
Mode of PMR invasion	
Invasion	17 (20.7)
Contact	12 (14.6)
Non-contact	53 (64.6)

*PMR* pterygomandibular raphe

### PMR invasion and its relationship to clinicopathological features

To determine the clinicopathological significance of PMR invasion, we conducted a univariate analysis. The results indicated that patients exhibiting contact- or invasion-type PMR invasion patterns were associated with endophytic clinical growth pattern (*p* < 0.001), advanced cT (*p* = 0.001) or cN (*p* < 0.001), advanced cStage (*p* < 0.001), endophytic clinical growth pattern (*p* < 0.001), lower differentiation tumor (*p* = 0.011), and high-grade POI (*p* = 0.009) in a statistically significant manner (Table 2). Conversely, there were no substantial differences in the age or sex of patients with different PMR invasion patterns (Table 2).

**Table 2:** Relationships between the mode of PMR invasion and clinicopathological features of squamous cell carcinoma of buccal mucosa

Characteristics	Total	Mode of PMR invasion			*p*-value
		Non-contact	Contact	Invasion	
		53	12	17	
Age					
< 64	21	4 (33.3)	6 (35.3)	11 (20.8)	0.393
≧ 65	61	8 (66.7)	11 (64.7)	42 (79.2)	
Sex					
Male	50	31 (58.5)	7 (58.3)	12 (70.6)	0.659
Female	32	22 (41.5)	5 (41.7)	5 (29.4)	
Clinical growth pattern					
Superficial	29	27 (50.9)	1 (8.3)	1 (5.9)	< 0.001**
Exophytic	27	17 (32.1)	5 (41.7)	5 (29.4)	
Endophytic	26	9 (17.0)	6 (50.0)	11 (64.7)	
cT					
1	12	11 (20.8)	1 (8.3)	0 (0.0)	< 0.001**
2	47	34 (64.2)	6 (50.0)	7 (41.2)	
3	8	4 (7.5)	4 (33.3)	0 (0.0)	
4	15	4 (7.5)	1 (8.3)	10 (58.8)	
cN					
0	47	40 (75.5)	4 (33.3)	3 (17.6)	< 0.001**
1	14	6 (11.3)	2 (16.7)	6 (35.3)	
2b	20	7 (13.2)	5 (41.7)	8 (47.1)	
2c	1	0 (0.0)	1 (8.3)	0 (0.0)	
cStage					
I	13	12 (22.6)	1 (8.3)	0 (0.0)	< 0.001**
II	29	24 (45.3)	3 (25.0)	2 (11.8)	
III	11	7 (13.2)	3 (25.0)	1 (5.9)	
IVA	29	10 (18.9)	5 (41.7)	14 (82.4)	
Differentiation					
Well	59	41 (77.4)	11 (91.7)	7 (41.2)	0.011*
Moderate	19	11 (20.8)	0 0.0)	8 (47.1)	
Poor	4	1 (1.9)	1 (8.3)	2 (11.8)	
Pattern of invasion					
POI-1	2	2 (3.8)	0 (0.0)	0 (0.0)	0.009**
POI-2	23	19 (35.8)	4 (33.3)	0 (0.0)	
POI-3	42	27 (50.9)	6 (50.0)	9 (52.9)	
POI-4	15	5 (9.4)	2 (16.7)	8 (47.1)	

Fisher’s exact test for categorical factors was used to calculate* p*-values

*PMR* pterygomandibular raphe, *POI* pattern of invasion

*p < 0.05, **p < 0.01

### Prognostic significance of PMR invasion in patients with SCCBM

**Fig. 1:**
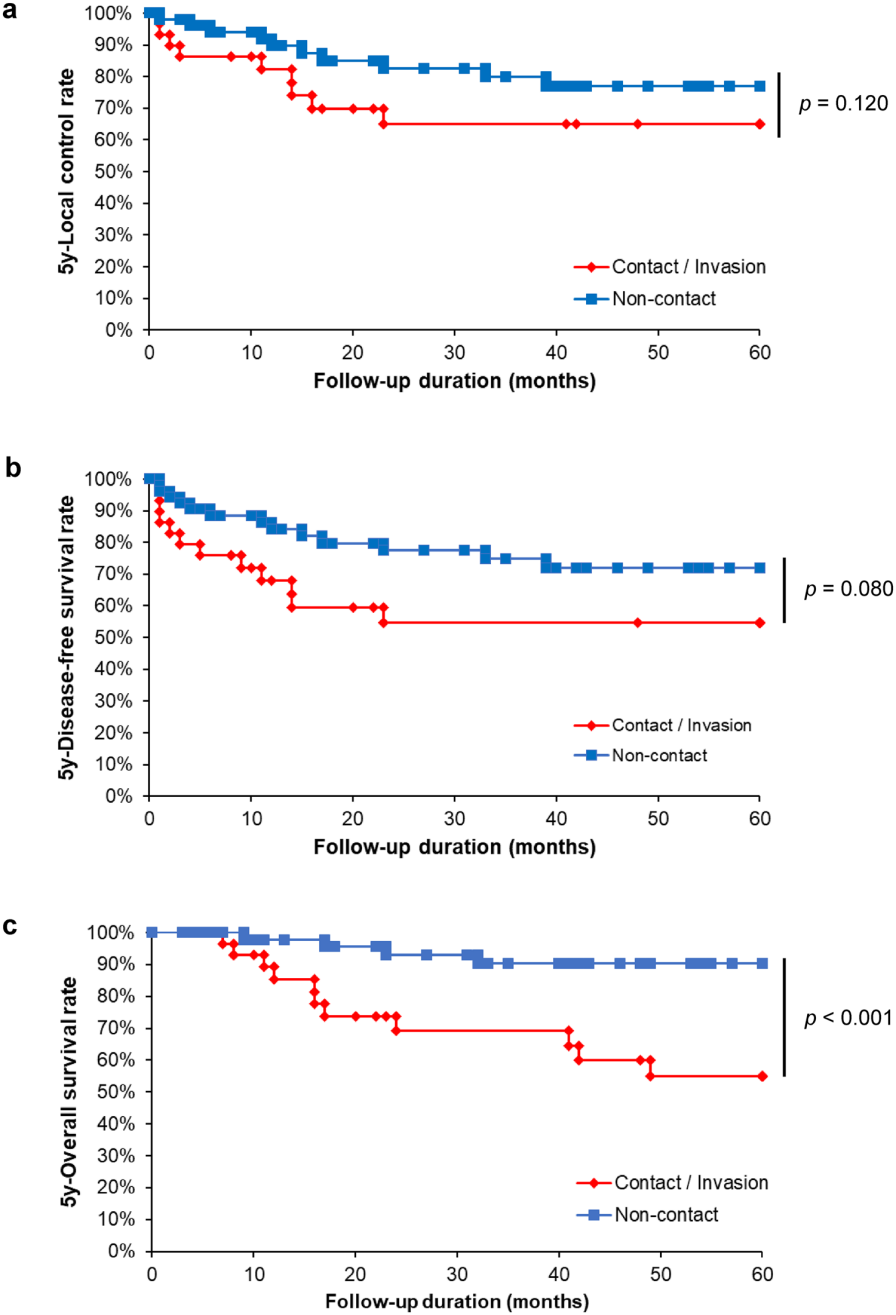
Prognostic impact of pterygomandibular raphe invasion on squamous cell carcinoma of the buccal mucosa. Kaplan–Meier analysis of 5-year LCR (**a**), DFS (**b**), and OS rates (**c**) in patients with SCCBM. LCR, local control rate; DFS, disease-free survival; OS, overall survival rate; PMR, pterygomandibular raphe. The patients were categorized into two groups based on the mode of PMR invasion

Because univariate analysis revealed a strong correlation between PMR invasion patterns and tumor malignancy, to determine whether PMR invasion affects the prognosis of patients with SCCBM, we examined the prognostic significance of PMR invasion using the log-rank test as a univariate analysis. As shown in Table 3, none of the variables showed prognostic significance for the 5-year LCR and DFS. In contrast, the presence of cN2b or higher (*p* = 0.035), moderate-to-poorly differentiated tumors (*p* = 0.027), POI-4 (*p* = 0.007), and PMR invasion of the contact or invasion type (*p* = 0.001) were significant predictors of OS. In a series of analyses, patients with contact- or invasion-type PMR invasion patterns demonstrated significantly worse prognoses in terms of OS but not in terms of LCR and DFS (Fig. 1a, b, and c).

**Table 3:** Univariate analysis of prognostic factors in 82 patients with squamous cell carcinoma of buccal mucosa

Variables	5y-LCR (%)	*p*-value	5y-DFS (%)	*p*-value	5y-OS (%)	*p*-value
Age						
< 64	79.2	0.666	82.7	0.410	75.4	0.989
≧65	72.4		73.6		76.6	
Sex						
Male	73.2	0.718	71.3	0.281	70.7	0.200
Female	75.1		82.2		84.6	
Clinical growth pattern						
Superficial/Exophytic	74.4	0.884	79.2	0.333	84.8	0.540
Endophytic	74.3		69.2		70.6	
cT						
cT1, 2, 3	77.9	0.195	77.8	0.398	82.9	0.084
cT4a	56.3		64.2		59.2	
cN						
cN0, 1	78.5	0.076	80.3	0.380	82.9	0.035*
cN2b, 2c	63.0		62.3		58.3	
cStage						
cStage I, II, III	77.5	0.307	79.5	0.554	86.5	0.041*
cStage IVA	68.7		69.0		60.0	
Differentiation						
Well	75.5	0.882	78.7	0.256	83.3	0.027*
Moderate, Poor	73.0		67.6		50.8	
Pattern of invasion						
POI-1, 2, 3	76.1	0.223	81.5	0.061	84.5	0.007**
POI-4	66.7		54.7		49.5	
Mode of PMR invasion						
Non-contact	79.2	0.120	83.3	0.080	90.4	0.001**
Contact/Invasion	65.9		62.7		54.9	

Survival analysis was performed using the Kaplan–Meier method and log-rank tests

*CI* confidence interval, *LCR* local control rate, *DFS* disease-free survival rate, *OS* overall survival, *PMR* pterygomandibular raphe

*p < 0.05, **p < 0.01

### Prognostic value of PMR invasion in SCCBM after PSM

**Fig. 2:**
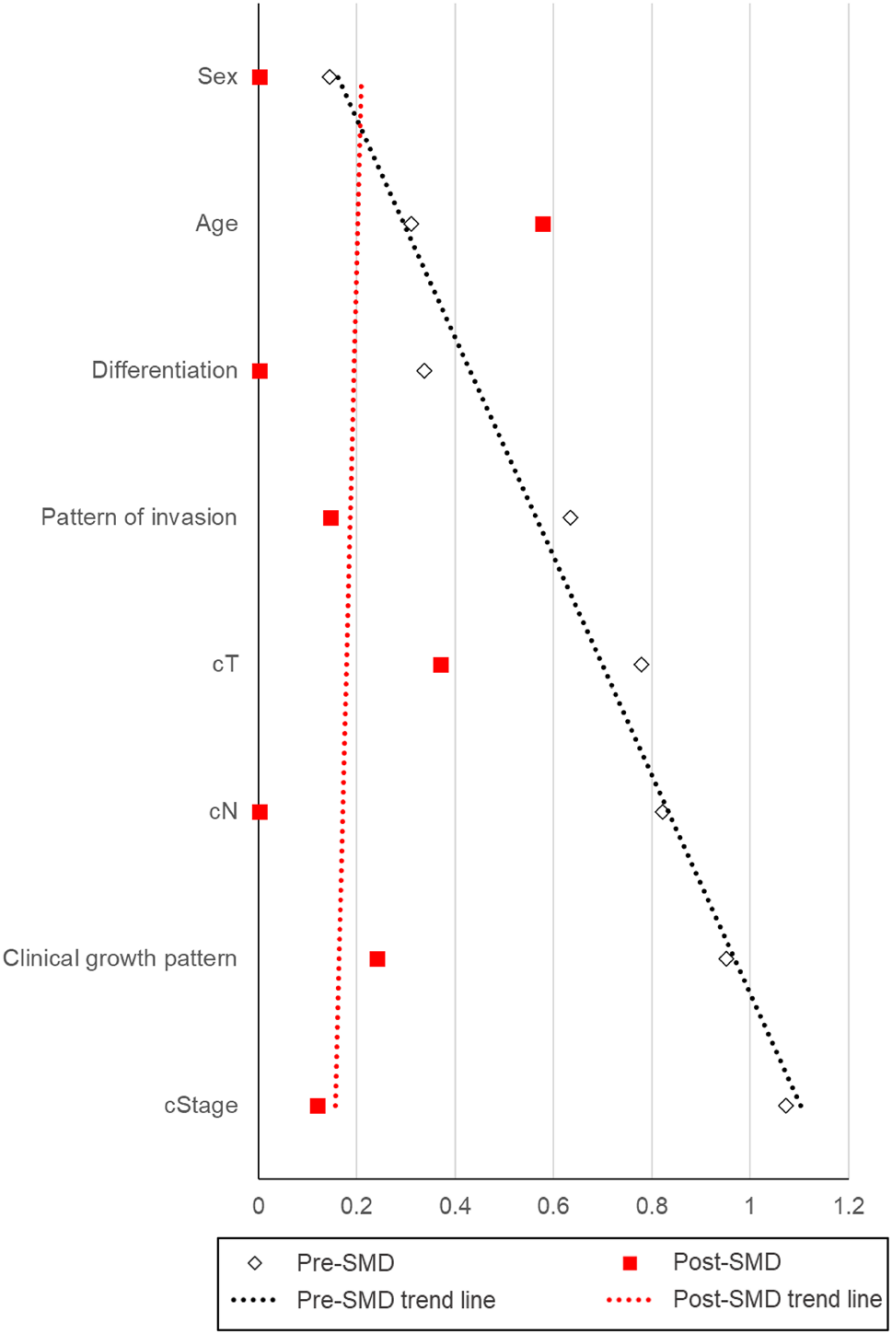
Pre- and post-match standardized mean differences (SMDs) for patient characteristics. The black diamonds indicate the standardized mean difference (SMD) before propensity score matching (PSM) (Pre-SMD). The black dashed line represents the approximate line of the SMD before the PSM analysis. The red rectangle denotes SMD after PSM (Post-SMD). The red dashed line represents the approximate line of the SMD following PSM analysis

**Fig. 3:**
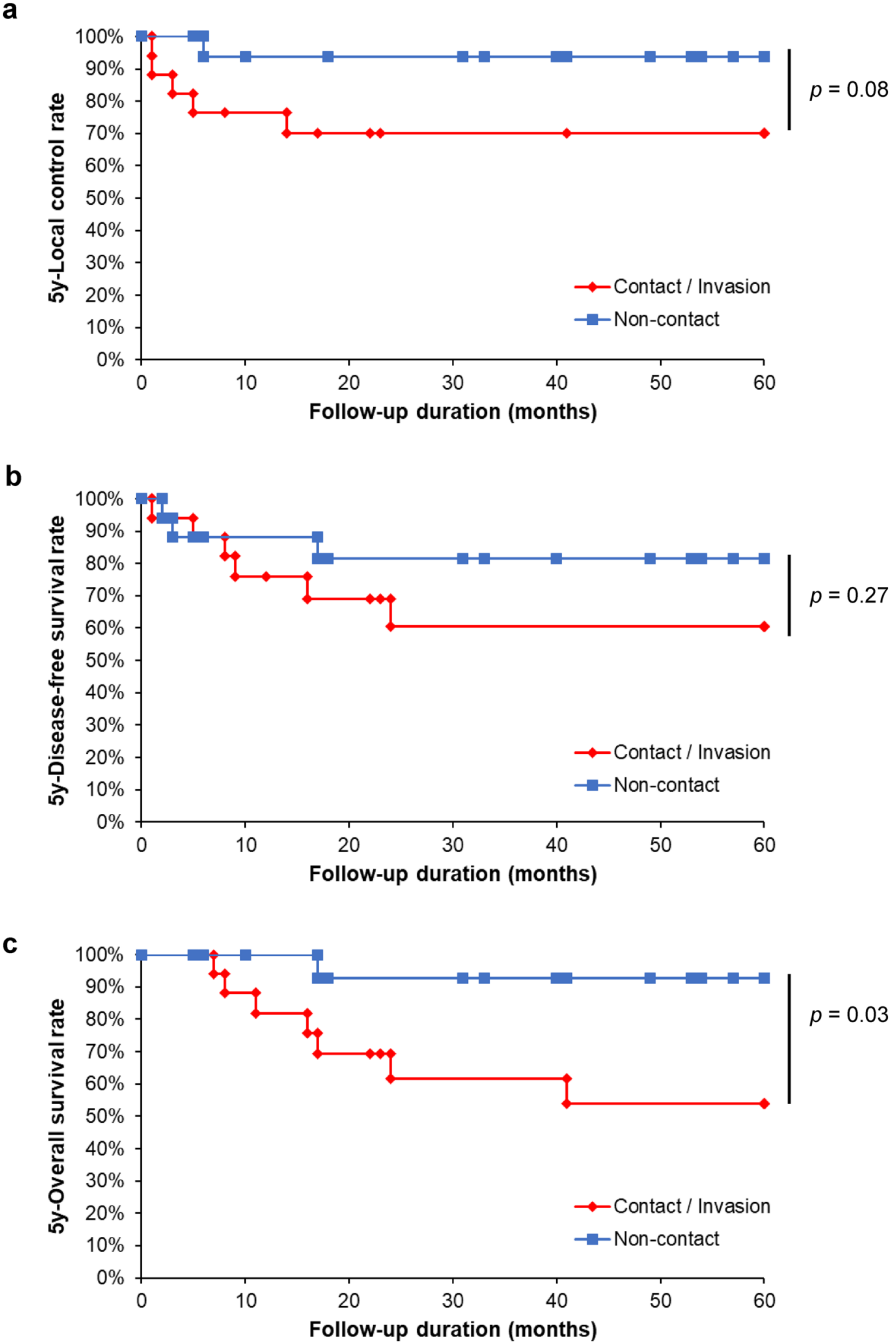
Prognostic impact of pterygomandibular raphe invasion on squamous cell carcinoma of the buccal mucosa after propensity score matching analysis. Kaplan–Meier analysis of 5-year LCR (**a**), DFS (**b**), and OS rates (**c**) in patients with SCCBM. LCR, local control rate; DFS, disease-free survival; OS, overall survival rate; PMR, pterygomandibular raphe. The patients were categorized into two groups based on the mode of PMR invasion

Because SCCBM is a rare OSCC subgroup, our retrospective analysis had a relatively small sample size. This longitudinal observational study is subject to numerous potential biases. In addition, there were significant differences in baseline characteristics between the non-contact and contact/invasion groups. Therefore, to elucidate the precise prognostic significance of PMR invasion, we conducted a PSM analysis. As shown in Table 4, each of the 17 patients was matched to the non-contact type and contact/invasion-type groups. For the post-match patient characteristics, no significant differences were observed between the two groups for any of the variables (Table 4). Moreover, most standardized mean difference (SMD) values were low post-matched in terms of patient characteristics, which addresses the efficacy of PSM in this study (Fig. 2). Following PSM, although there was no statistical significance, the LCR and DFS in the contact/invasion group were lower than those in the non-contact group (Fig. 3a and b, *p* = 0.08 and 0.27, respectively).In contrast, we confirmed that the 5-year OS of the contact/invasion group was significantly poorer than that of the non-contact group (Fig. 3c, *p* = 0.03).

**Table 4:** Baseline charcteristics of patients with SCCBM brfore and after PSM

		Before matching			After matching		
		Non-contact	Contact/Invasion	*p*-value	Non-contact	Contact/Invasion	*p*-value
Caracteristics	Groups	53	29		17	17	
Age	≦ 64	11 (20.8)	10 (34.5)	0.194	2 (11.8)	6 (35.3)	0.225
	≧ 65	42 (79.2)	19 (65.5)		15 (88.2)	11 (64.7)	
Sex	Male	31 (58.5)	19 (65.5)	0.638	10 (58.8)	10 (58.8)	1
	Female	22 (41.5)	10 (34.5)		7 (41.2)	7 (41.2)	
Clinical growth pattern	Endophytic	9 (17.0)	17 (58.6)	< 0.001**	8 (47.1)	6 (35.3)	0.728
	Superficial/Exophytic	44 (83.0)	12 (41.4)		9 (52.9)	11 (64.7)	
cT	1, 2, 3	45 (84.9)	14 (48.3)	0.001	16 (94.1)	14 (82.4)	0.601
	4a	8 (15.1)	15 (51.7)		1 (5.9)	3 (17.6)	
cN	0, 1	46 (86.8)	15 (51.7)	0.001	11 (64.7)	11 (64.7)	1
	2b, 2c	7 (13.2)	14 (48.3)		6 (35.3)	6 (35.3)	
cStage	I, II, III	36 (67.9)	6 (20.7)	< 0.001**	10 (58.8)	9 (52.9)	1
	IVA	17 (32.1)	23 (79.3)		7 (41.2)	8 (47.1)	
Differentiation	Poor, Moderate	12 (22.6)	11 (37.9)	0.198	5 (29.4)	5 (29.4)	1
	Well	41 (77.4)	18 (62.1)		12 (70.6)	12 (70.6)	
Pattern of invasion	POI-1, 2, 3	48 (90.6)	19 (65.5)	0.008**	14 (82.4)	13 (76.5)	1
	POI-4	5 (9.4)	10 (34.5)		3 (17.6)	4 (23.5)	

Propensity score matching was conducted to equate background variables between the non-contact and contact/invasion groups

Fisher’s exact test for categorical factors was used to calculate *p*-values between mode of PMR invasion and clinicopathological features

*SCCBM* squamous cell carcinoma of buccal mocosa, *PSM* propensity score-matching, *POI* pattern of invasion

^**^p < 0.01

### Examination of the impact of PMR invasion on recurrence rates and control outcomes

During post-treatment follow-up, 17 patients (20.7%) experienced local failure, 4 (4.9%) developed delayed neck metastasis, and 6 (7.3%) exhibited distant metastasis. The correlation analysis based on the Kapan–Meier method between PMR invasion and clinical course revealed that the contact/invasion-type group demonstrated a significantly worse distant metastasis-free survival (DMFS) (*p* = 0.011; Table 5). Although a trend toward poorer prognosis was observed, there was no statistically significant difference in LCR or delayed neck metastasis-free survival between groups (*p* = 0.120 and *p* = 0.067, respectively; Table 5). A comprehensive analysis of recurrent cases was performed to investigate the influence of PMR invasion on local recurrence and operative outcomes. Among the 82 patients, 17 (20.7%) experienced local recurrence, including 9 non-contact, 4 contact, and 4 invasion types. The local recurrence rates for these categories were 17.0%, 33.3%, and 23.5%, respectively, with contact and invasion types demonstrating higher local recurrence rates. Furthermore, a detailed assessment of the 17 recurrence cases indicated that 10 cases involved superficial recurrence in the buccal mucosa, oral floor, palate, and lower gingiva; whereas, 7 cases involved deep recurrence in the buccal subcutaneous, mandible, and masticator space, among other areas. Analyzing the recurrence pattern using PMR invasion, it was observed that the invasion type demonstrated a significantly higher rate of deep recurrence. (*p* = 0.032; Table 6). Furthermore, the cumulative LCR after salvage operation diminished as the invasion type worsened (60.0%, 37.5%, and 25.0% for non-contact, contact, and invasion patterns, respectively; *p* = 0.651; Table 7). Furthermore, an exploratory study investigated the associations between PMR invasion mode, high-risk factors for postoperative recurrence or metastasis, and postoperative adjuvant therapy. The results indicated a significantly higher proportion of ENE in the invasion type (*p* = 0.011). The incidence of positive surgical margins was higher in the invasion-type group. Consequently, among the 11 patients with high-risk factors for postoperative recurrence or metastasis, all 4 patients receiving postoperative adjuvant therapy were of the invasion type, which was significantly higher than other types (*p* < 0.001; Table 8).

**Table 5:** Impact of PMR invasion on clinical course in 82 patients with squamous cell carcinoma of buccal mucosa

Variables		5y-LCR (%)	*p*-value	5y-DNMFS (%)	*p*-value	5y-DMFS (%)	*p*-value
Mode of PMR invasion							
Non-contact	n = 53	79.2	0.120	96.6	0.067	97.7	0.011*
Contact/Invasion	n = 29	65.9		94.3		81.7	

Survival analysis was performed using the Kaplan–Meier method and log-rank tests

*CI* confidence interval, *LCR* local contro rate, *DNMFS* delayed neck metastasis-free survival, *DMFS* distant metastasis-free survival, *PMR* pterygomandibular raphe

*p < 0.05

**Table 6:** Relationships between the mode of PMR invasion and local recurrence rate and pattern

Cases of local recurrence (rate)		Mode of PMR invasion			*p*-value
	Total	Non-contact	Contact	Invasion	
	82	53	12	17	
	17 (20.7%)	9 (17.0%)	4 (33.3%)	4 (23.5%)	
Recurrence pattern		n = 9	n = 4	n = 4	
Superficial	10	6 (66.7%)	4 (100%)	0 (0.0%)	0.032*
Deep	7	3 (33,3%)	0 (0.0%)	4 (100%)	

Fisher’s exact test for categorical factors was used to calculate *p*-values between mode of PMR invasion and recurrence patterns

*PMR* pterygomandibular raphe, *Superficial* buccal mucosa, oral floor, palate, lower gingiva, *Deep* buccal subcutaneous, mandible, masticator space

^*^p < 0.05

**Table 7:** Relationships between the mode of PMR invasion and cumulative local control rate after salvage operation in recurrence cases

		Mode of PMR invasion			*p*-value
	Total	Non-contact	Contact	Invasion	
Number of case	n = 17	n = 9	n = 4	n = 4	
Cumulative local control rate (%)		60.0	37.5	25.0	0.651

Cumulative local control rate was examined based on the Kaplan–Meier method and log-rank tests

*PMR* pterygomandibular raphe

**Table 8:** Relationships between the mode of PMR invasion, high-risk factors for postoperative recurrence or metastasis and the administration of postoperative adjuvant therapy

Chatacteristics		Mode of PMR invasion			*p*-value
	Total	Non-contact	Contact	Invasion	
Number of case	82	53	12	17	
Positive surgical margin					
Absent	75	48 (90.6)	11 (91.7)	16 (94.1)	0.901
Present	7	5 (9.4)	1 (8.3)	1 (5.9)	
Extranodal extension					
Absent	78	53 (100.0)	11 (91.7)	14 (82.4)	0.011*
Present	4	0 (0.0)	1 (8.3)	3 (17.6)	
Postoperative adjuvant treatment					
No	78	53 (100.0)	12 (100.0)	13 (76.5)	< 0.001**
Yes	4	0 (0.0)	0 (0.0)	4 (23.5)	

Fisher’s exact test for categorical factors was used to calculate *p*-values

*PMR* pterygomandibular raphe

^*^p < 0.05, ^**^p < 0.01

## Discussion

This study revealed that SCCBM clinicopathological characteristics were primarily observed in patients aged ≥ 65 years, with most cases classified as cT1-2. Metastases were clinically diagnosed in approximately 50% of the patients. Except in specific Asian regions, where betel nuts cause it [7], SCCBM is typically 5%–10% [5], and in Japan, approximately 7.1% [9]. Recent Japanese studies on SCCBM are limited, such as those conducted by Hirai et al. [17] and Kugimoto et al. [18]. Our study showed no substantial variation in clinicopathological characteristics compared with these findings. PMR is an important anatomical indicator in patients with posterior extension of oral cancers. Although Otsuru et al. [11] examined PMR invasion in OSCC, no study has specifically analyzed the PMR invasion pattern in SCCBM. This is the first study to ascertain the frequency of PMR invasion in SCCBM. Notably, the rate of cases with PMR invasion in SCCBM (20.7%) was close to the proportion of cases classified as invading the retromolar area when focusing on SCCBM (approximately 20%) [18], suggesting that a certain proportion of tumors invaded the retromolar area during PMR invasion.

The present study revealed that clinically advanced PMR invasion patterns were correlated with cT and cN as well as with various factors associated with malignant tumor phenotypes, such as endophytic invasion, high-grade POI, and distant metastasis. In a previous study, Otsuru et al. reported that more severe PMR invasion was associated with advanced T stage, positive histological lymph node metastasis, and poorly differentiated tumors [11]. This implies that patients with highly invasive SCCBM are more susceptible to PMR invasion. Furthermore, distant metastasis may be associated with the highly invasive and metastatic potential of SCCBM, which increases the tendency for PMR invasion. Therefore, the presence or absence of PMR invasion should be carefully considered when determining the extent of resection and postoperative adjuvant therapy for patients with highly invasive SCCBM. Because PMR is contiguous with various anatomical structures, including the mandible, masticator space, mesopharynx, and maxilla, PMR invasion is likely to lead to invasion into the masticator space [3]. Hence, although T4b cases were completely excluded from the current analysis, the presence or absence of PMR invasion should be considered in the initial diagnosis, as cases with risks comparable to T4b cases with challenges in local control may have been included. Furthermore, considering that PMR invasion was associated with various adverse SCCBM characteristics, including invasiveness and distant metastasis, even without progression to T4b stage in this study, PMR invasion should be regarded as a novel grading index for SCCBM. Thus, our findings indicated that PMR invasion should be regarded as a novel indicator of malignancy in buccal mucosal lesions.

The PMR invasion pattern of the contact or invasion type was identified as a prognostic factor for the 5-year OS in this study. Notably, the effect of PMR invasion on 5-year OS was maintained after PSM. This is the first study to clarify the prognostic significance of PMR invasion pattern in resectable SCCBM. Otsuru et al. reported PMR invasion as an independent prognostic factor for LCR, OS, and DSS in all OSCC subtypes [11]. However, their study analyzed all oral subsites and was not limited to SCCBM. Several studies have identified multiple prognostic factors for SCCBM. According to Bachar et al., ENE is an independent prognostic factor for 5-year OS in patients with SCCBM [19]. Similarly, Marineli et al. reported that advanced age, POI, and PNI were independent prognostic factors for the 5-year OS [9]. Furthermore, Niu et al. discovered pathological cervical lymph node status as an independent prognostic factor for 3-year disease-specific survival [20]. However, these reports did not incorporate the PMR invasion patterns. In the present study, the PMR invasion pattern, cN status, cStage, differentiation, and POI were identified as prognostic factors. The prognostic value of PMR invasion was retained for the 5-year OS after PSM. Therefore, the present study indicates that PMR invasion is a useful prognostic factor that is comparable to the existing factors. Furthermore, PMR invasion demonstrated a significant correlation with ENE and distant metastasis. This observation indicates the potential use of PMR invasion as a prognostic indicator for assessing the grade of SCCBM. However, notably, this case series study was conducted over an extended period, during which postoperative treatment administration exhibited variability. Future research should focus on assessing the efficacy of adjuvant therapy in patients with PMR invasion within a larger cohort, using a standardized treatment protocol, and considering existing postoperative risk factors. The inclusion of PMR invasion pattern as a novel risk factor for SCCBM could facilitate the development of therapeutic methods for this difficult-to-treat region.

Unlike the findings of Otsuru et al. [11], the results of this study did not identify PMR invasion as a significant prognostic indicator for LCR or DFS in either the entire or the PSM cohort. This difference can be interpreted in several ways. Earlier studies included all OSCC cases and some cT4b cases in their analysis. Moreover, the decision to perform extensive resection involving the maxilla, mandible, pterygoid muscle, and mesopharynx in patients with PMR invasion may have lowered the recurrence rate associated with PMR invasion. Concerning local recurrence, patients with the non-contact type demonstrated a higher superficial recurrence rate, likely due to field carcinogenesis or other factors. No significant difference was observed in the occurrence of delayed neck metastases, possibly because elective neck dissection was conducted during reconstructive surgery in patients undergoing extensive resection. These elements may explain the absence of a significant difference in LCR or DFS between patients with non-contact and contact/invasion types. Conversely, the contact/invasion group demonstrated a notably higher distant metastasis rate, which may have significantly contributed to the decrease in OS.

In the present study, PMR invasion of the deep recurrence type was high in the buccal subcutaneous, mandible and masticator space, with a low rate of surgical control over recurrent lesions. This could be attributed to the contiguity of the PMR with various adjacent anatomical structures and its role in connecting the superficial and deep portions of the oral cavity [3, 11]. SCCBM with an invasion-type PMR invasion pattern is often a highly invasive carcinoma that may rapidly infiltrate into the medial pterygoid muscle and mandibular cortical bone. Otsuru et al. emphasized establishing a sufficient safety margin in the buccinatopharyngeus muscle fascia during resection of OSCC with posterior extension [11]. Based on these findings and previous research, removal of both the mandible and medial pterygoid muscle with a mandibular swing approach [21] and contiguous anatomic structures with PMR should be considered in patients with SCCBM and PMR involvement, even without apparent mandibular or masticator space involvement. However, our study focused on patients with resectable SCCBM whose preoperative MRI images were available. Additional studies of unresectable SCCBM are required to determine the true frequency of PMR invasion. The oncological differences between T4b stage tumors and invasion-type PMR invasion pattern tumors that do not progress to stage T4b should be examined.

Several factors should be considered when interpreting these results. This was a single-center retrospective analysis, with a limited sample size. The analysis encompassed an extended period (18 years), during which diagnostic imaging accuracy may have been influenced by variations in the MR equipment specifications. To address these limitations, a more rigorously controlled, prospective, multicenter observational study is necessary. Furthermore, in this study, we conducted a retrospective analysis and did not consider PMR during the preparation of pathological specimens from the resected tumors; thus, pathologically confirming the presence or absence of PMR invasion was challenging. Future research is warranted to investigate the clinical significance of pathological PMR invasion in SCCBM with respect to its potential role as a pre-treatment indicator.

In conclusion, we identified the clinical characteristics of resectable SCCBM and demonstrated that PMR invasion may serve as a novel grading and prognostic factor for SCCBM, excluding stage T4b tumors. We also showed that PMR invasion may be significant for planning the extent of SCCBM resection. Despite the limitations of the present study, the PMR invasion pattern can be employed as a grading system for SCCBM tumors and to develop future treatment strategies.

## Supplementary Information

Below is the link to the electronic supplementary material.

Supplementary file1 (PDF 217 KB)

## Data Availability

The datasets used and/or analyzed during the current study are available from the corresponding author upon reasonable request.
