# Supplementary material for: Pterygomandibular raphe invasion as a novel grading and prognostic factor for squamous cell carcinoma of buccal mucosa: a retrospective study with propensity score matching
Source: Int J Clin Oncol. 2025 Jul 11;30(9):1775–86. doi: 10.1007/s10147-025-02821-2 (PMC12378123; doi:10.1007/s10147-025-02821-2)

Supplementary Fig. S1 Pterygomandibular raphe invasion pattern (enhanced MRI)

A. Non-contact type. White arrow: Tumor; Yellow triangle: pterygomandibular raphe.

B. Contact type. White arrow: Tumor; Yellow triangle: pterygomandibular raphe.

C. Invasion type. White arrow: Tumor; Yellow triangle: pterygomandibular raphe.

Supplementary Fig. S1

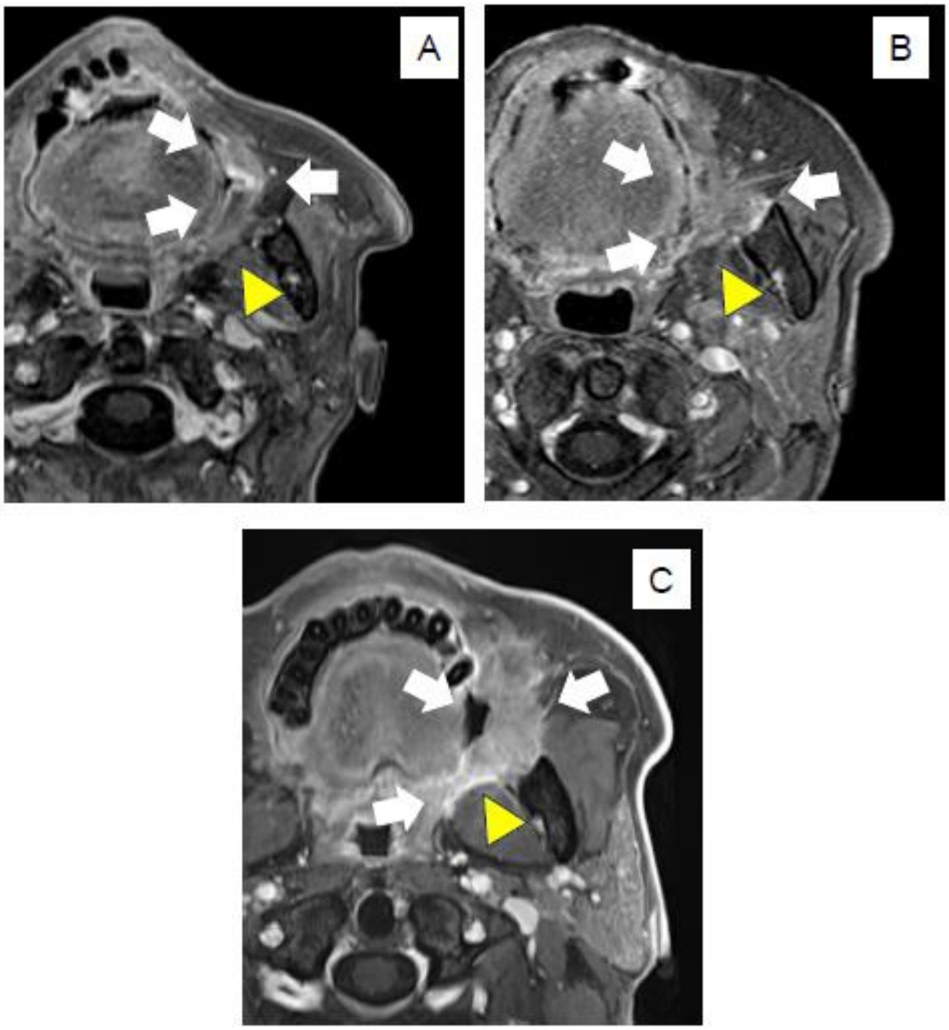

Supplement: Supplementary file 1 — Supplementary file1 (PDF 217 KB) [file 10147_2025_2821_MOESM1_ESM.pdf]
